# The Dimethylsulfide Cycle in the Eutrophied Southern North Sea: A Model Study Integrating Phytoplankton and Bacterial Processes

**DOI:** 10.1371/journal.pone.0085862

**Published:** 2014-01-17

**Authors:** Nathalie Gypens, Alberto V. Borges, Gaelle Speeckaert, Christiane Lancelot

**Affiliations:** 1 Ecologie des Systèmes Aquatiques, Université Libre de Bruxelles, Brussels, Belgium; 2 Unité d'Océanographie Chimique, Université de Liège, Liège, Belgium; Mount Allison University, Canada

## Abstract

We developed a module describing the dimethylsulfoniopropionate (DMSP) and dimethylsulfide (DMS) dynamics, including biological transformations by phytoplankton and bacteria, and physico-chemical processes (including DMS air-sea exchange). This module was integrated in the MIRO ecological model and applied in a 0D frame in the Southern North Sea (SNS). The DMS(P) module is built on parameterizations derived from available knowledge on DMS(P) sources, transformations and sinks, and provides an explicit representation of bacterial activity in contrast to most of existing models that only include phytoplankton process (and abiotic transformations). The model is tested in a highly productive coastal ecosystem (the Belgian coastal zone, BCZ) dominated by diatoms and the Haptophyceae *Phaeocystis*, respectively low and high DMSP producers. On an annual basis, the particulate DMSP (DMSPp) production simulated in 1989 is mainly related to *Phaeocystis* colonies (78%) rather than diatoms (13%) and nanoflagellates (9%). Accordingly, sensitivity analysis shows that the model responds more to changes in the sulfur:carbon (S:C) quota and lyase yield of *Phaeocystis*. DMS originates equally from phytoplankton and bacterial DMSP-lyase activity and only 3% of the DMS is emitted to the atmosphere. Model analysis demonstrates the sensitivity of DMS emission towards the atmosphere to the description and parameterization of biological processes emphasizing the need of adequately representing in models both phytoplankton and bacterial processes affecting DMS(P) dynamics. This is particularly important in eutrophied coastal environments such as the SNS dominated by high non-diatom blooms and where empirical models developed from data-sets biased towards open ocean conditions do not satisfactorily predict the timing and amplitude of the DMS seasonal cycle. In order to predict future feedbacks of DMS emissions on climate, it is needed to account for hotspots of DMS emissions from coastal environments that, if eutrophied, are dominated not only by diatoms.

## Introduction

Dimethylsulfide (DMS) is a volatile sulfur (S) compound that plays an important role in the global S cycle and may control climate by influencing cloud albedo through the emission of atmospheric aerosols [1]. However, the significance of this feedback remains uncertain [2], as the present knowledge of mechanisms controlling DMS production is insufficient to allow a realistic description of DMS(P) production in Earth System models [3], and predict with confidence the impact of future climate change on surface ocean DMS [4], [5], [6].

In marine ecosystems, phytoplankton are the primary producers of dimethylsulfoniopropionate (DMSP), the precursor of the DMS (e.g. [7]). However, the amount of DMSP synthesized by cells varies among phytoplankton classes and species [8], [9], as well as with the physiological status [10], [11]. Overall, Bacillariophyceae (diatoms) synthesize less DMSP than Dinophyceae and Haptophyceae [8]. The metabolical role of DMSP in marine organisms is still unclear [7]. DMSP has been suggested to play a role as an osmoprotectant [12], [13], as a cryoprotectant [14], [15], [16], and as a nitrogen salvage mechanism during growth limitation [11], [17]. The DMS and/or acrylic acid derived from DMSP cleavage might also act for phytoplankton as an antioxidant [18], [19], [20], as a deterrent for zooplankton [21], [22], [23], or as an anti-viral [24]. The conversion of DMSP to DMS and acrylic acid is catalysed by phytoplankton DMSP-lyases [25]. The intracellular DMSP is also released in the water column as dissolved DMSP (DMSPd) through various phytoplankton mortality processes, including cell lysis [26], [27], grazing pressure [22], [28], and viral infection [29]. Once in the water column, DMSPd is available for assimilation and degradation by bacterioplankton and part of the DMSPd is cleaved into DMS through bacterial metabolism [30], [31], [32]. Although largely variable, phytoplankton and bacterial lyases might contribute almost equally to the DMS production in marine ecosystems [7], [33], [34]. Yet, the main part of DMSPd is degraded by bacteria through the demethylation/demethiolation pathways for fulfilling their S and/or carbon (C) needs [35]. Once produced, DMS can also be consumed by bacteria to satisfy S and mainly C needs [36], photooxided [37], [38], or emitted to the atmosphere across the air-sea interface [39], [40]. The relative importance of these processes is variable and depends on physical forcing factors, but observational evidence suggests that microbial consumption and photooxidation are the main DMS fates [38], [41], [42]. Because DMS production results from the balance of several complex processes, the link between DMSP production and atmospheric DMS emission is not direct and statistical relationships between DMS concentrations and other environmental variables (such as chlorophyll a (Chl *a*), nutrients, irradiance or mixed layer depth) are uncertain and generally regional in scope [39], [40].

Several mechanistic models of different biological complexity (reviewed by Le Clainche et al. [43]) have been therefore developed to better assess and understand DMS production and controlling factors in marine ecosystem [44], [45], [46], [47], [48], [49], [50], [51], [52], [53], [54], [55], [56], [57], [58]. All these models couple a biogenic S module composed of two or three state variables (DMS, particulate DMSP (DMSPp) and/or DMSPd) to a C- or nitrogen- (N) based ecological model of the plankton community [43], [59]. Most of them subdivide phytoplankton into several functional groups characterized by a specific DMSP cell quota (S:C) in agreement with observations [7]. S:C quota is generally considered as a constant with the exception of models of Le Clainche et al. [52] and Polimene et al. [58] that include variation of S:C with light intensity. The representation of heterotrophic compartments is generally less complex [43] and only some recent modelling studies include an explicit representation of the bacteria (e.g. [50], [56], [57], [58]). To the best of our knowledge the DMSP/DMS model of Archer et al. [50] is the only attempt to link the DMSP/DMS fate to bacterial degradation of organic matter, distinguishing between C and DMS- and DMSP-consuming bacteria types. These authors conclude that a tight coupling between the ecological processes and the DMS cycle is required to properly model DMS emissions to the atmosphere due to both the species dependence of DMSP production and the complexity of microbial metabolic pathways leading to the production of DMS.

Accordingly, we integrated a module describing the DMS(P) cycle into the existing ecological MIRO model [60] that describes C and nutrients cycles in the Southern North Sea (SNS) with an explicit description of the phytoplankton and bacteria dynamics to study the microbial controls of DMS(P) production and fate including DMS emission to the atmosphere. The MIRO model is a conceptual model of the biogeochemical functioning of marine ecosystem that includes an explicit description of growth and fate of *Phaeocystis* (Haptophyceae) that is one of the most intense DMSP producers [8], [61], [62]. The model was applied to the English Channel and the SNS with a focus to the Belgian coastal waters characterized by massive spring blooms of *Phaeocystis globosa* that develops between the spring and summer diatom blooms (e.g. [63], [64], [65]) in response to excess NO_3_^−^ river inputs [66]. This is an adequate case study of *Phaeocystis*-dominated coastal area where the model can be applied to study the link between DMSP production/cleavage by phytoplankton, DMS(P) bacterial transformation, and DMS emissions as field observations also report important DMS concentration [33], [67], [68]. The NE Atlantic Shelves (including the SNS) were indeed pointed as “hot-spot” areas for DMS concentrations (with the Atlantic Subarctic region) in the Atlantic Ocean [40].

In this paper, we first describe the concepts behind the DMS(P) mathematical model and its coupling with the ecological MIRO model (MIRO-DMS). The model is then applied in the SNS to describe the seasonal evolution of DMS(P) and the associated DMS emission to the atmosphere, and provide an annual budget of DMS(P) fluxes. Sensitivity tests on parameters are conducted to identify key microbial controls of DMS(P) production and how these change the emission of DMS to the atmosphere. Finally, we test the applicability of several published empirical relationships that predict DMS from other variables such as Chl *a*.

## Materials and Methods

### Model description

The MIRO-DMS model results from the coupling between a module describing the DMS(P) dynamics and the existing ecological MIRO model developed to represent the dynamics of the ecosystem of the North Sea dominated by *Phaeocystis* colonies [60], [69].

The ecological MIRO model, describing C, N, phosphorus (P) and silica (Si) cycles, assembles four modules describing the dynamics of three phytoplankton Functional Types (FT; diatoms, nanoflagellates and *Phaeocystis* colonies), two zooplankton FT (meso- and microzooplankton) and one bacteria FT involved in the degradation of dissolved and particulate organic matter (each with two classes of biodegradability) and the regeneration of inorganic nutrients (NO_3_^−^, NH_4_^+^, PO_4_^3−^ and Si(OH)_4_) in the water column and the sediment. Equations and parameters were formulated based on current knowledge of the kinetics and the factors controlling the main auto- and heterotrophic processes involved in the functioning of the coastal marine ecosystem (fully documented by Lancelot et al. [60] and in http://www.int-res.com/journals/suppl/appendix_lancelot.pdf).

**Figure 1:**
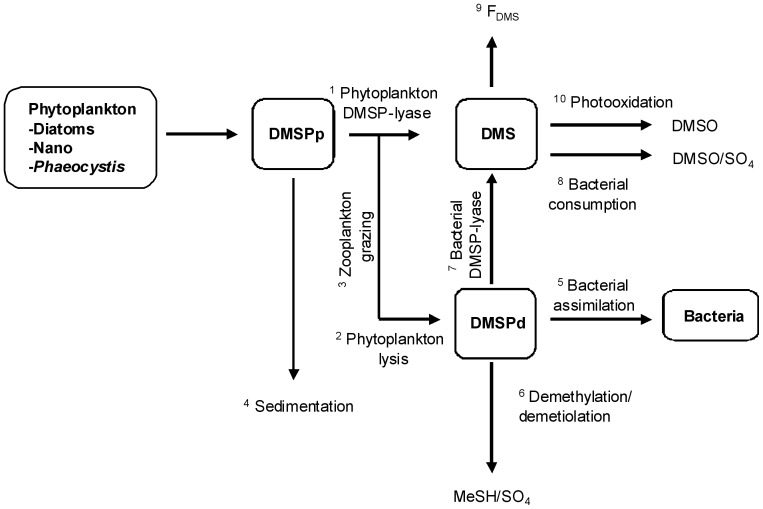
Diagram representing the state variables and processes of the DMS cycle incorporated into the ecological MIRO model.

The description of the DMS cycle requires the addition of three state variables: DMSPp associated to phytoplankton cells, DMSPd and DMS. Processes and parameters describing the DMS(P) cycle (Fig. 1) and its link with carbon rates in MIRO are described below by equations 1 to 12.

#### DMSPp synthesis and fate

The DMSPp is a constitutive compatible solute produced by phytoplankton cell [11]. In the MIRO-DMS model, the DMSPp cellular production and fate are similar to those of other phytoplankton functional molecules, with DMSPp production linked to phytoplankton growth, and DMSPp loss mainly resulting from cell lysis, micro/mesozooplankton grazing and sedimentation (Eq. 1). These processes are described for each phytoplankton FT (diatoms (DA), nanoflagellates (NF) and *Phaeocystis* colonies (OP) expressed in mgC m^−3^) as in the MIRO model and a specific DMSP:C quota (*SC*) is attributed to the three phytoplankton types. The DMSPp (in mmolS m^−3^) state equation is:



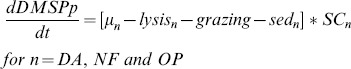
(1)


where *μ_n_* represents the growth of different phytoplankton types (in mgC m^−3^ h^−1^), *lysis_n_* is the phytoplankton lysis (in mgC m^−3^ h^−1^) (flux_1+2_, Fig. 1) and *SC_n_* is the intracellular phytoplankton S:C quotas (molS:mgC) derived from the literature (Table 1; [7]). *sed_n_* correspond to the loss of DMSPp due to diatoms and *Phaeocystis* colonies sedimentation (in mgC m^−3^ h^−1^) (flux_4_, Fig. 1). In the model, the sedimentation of nanoflagellates is considered as null. *grazing* is the predation pressure of micro and mesozooplankton on respectively on nanoflagellates (NF) and diatoms (DA) (in mgC m^−3^ h^−1^) (flux_3_, Fig. 1). *Phaeocystis* colonies (OP) are not subject to grazing [70].

**Table 1:** DMS(P) model parameters.

Parameter	Description	Units	Value	Reference
***SC_DA_***	Diatoms S:C quota	molS:mgC (molS:molC)	0.000072 (0.00086)	Stefels et al. [7]
***SC_NF_***	Nanoflagellates S:C quota	molS:mgC (molS:molC)	0.00092 (0.011)	Stefels et al. [7]
***SC_OP_***	*Phaeocystis* colonies S:C quota	molS:mgC (molS:molC)	0.00092 (0.011)	Stefels et al. [7]
***SC_BC_***	Bacteria S:C quota	molS:molC	0.01	Fagerbakke et al. [103]
***y^DA^_DMS_***	Part of diatoms DMSPp hydrolysed in DMS by phytoplankton lyase	-	0.1	Niki et al. [34]
***y^NF^_DMS_***	Part of nanoflagellates DMSPp hydrolysed in DMS by phytoplankton lyase	-	0.1	Niki et al. [34]
***y^OP^_DMS_***	Part of *Phaeocystis* colonies DMSPp hydrolysed in DMS by phytoplankton lyase	-	0.1	Niki et al. [34]
***K0***	Sea surface photooxydation rate	h^−1^	0.09	Brugger et al. [91]
***Ratio^BC^_S_***	Bacteria ratio using DMS(P) as substrate for sustain their S need	-	1	

#### DMSPd release and fate

The DMSPd simulated in the water column results from the DMSPp released after phytoplankton lysis and zooplankton grazing. When released, DMSPp remains partly as DMSPd in the water column but is also partly directly cleaved in DMS by phytoplankton DMSP-lyases [22], [25], [61], [71], [72], [73]. The DMSPd originated from micro-and meso-zooplankton grazing is either directly released by “sloppy-feeding”, excretion or egestion [21] and can represent up to 70% of the ingested DMSPp [74]. Wolfe and Steinke [22] also suggested that part of the DMSPp is directly converted to DMS. In the model, we assume that all the DMSPp ingested by micro- and meso-zooplankton is transformed into DMSPd (Eq. 1, 2). The fate of DMSPd is controlled by bacteria either through enzymatic cleavage into DMS and/or by demethylation/demethiolation, i.e. the cleavage of DMSPd to methanethiol (MeSH) [75] and acrylate or propionate [76] for fulfilling the C and S needs of bacteria [77], [78]. In the model, the state equation of DMSPd (in mmolS m^−3^) is:
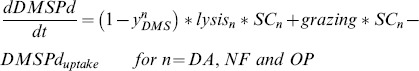
(2)where 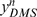 corresponds to the fraction of DMSPp directly cleaved in DMS by phytoplankton DMSP-lyases. In the reference simulation, this fraction was set to 10% of the DMSPp released for each phytoplankton group [34]. *lysis_n_* (flux_2_, Fig. 1) and *grazing* (flux_3_, Fig. 1) respectively are the phytoplankton cellular lysis and grazing (in mgC m^−3^ h^−1^) and *DMSPd_uptake_* is the bacterial uptake of DMSPd (in mmolS m^−3^ h^−1^) (flux_5+6+7_, Fig. 1).

The description of *DMSPd_uptake_* is based on the bacterial C uptake described in MIRO, adjusted with the DMSPd stoichiometry of C substrates available to bacteria and taking consideration of the proportion of the bacterial community using the DMSPd (and DMS if necessary) for their C and S needs: 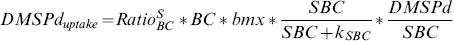
(3)where 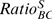 is the proportion of the bacterial community using the DMSPd for their S and C need. As a first approximation, we consider in the model that the whole bacterial community is able to degrade DMSP (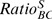 = 1). *BC* is the bacterial biomass, *bmx* is the bacterial growth, *SBC* are monomeric C substrates available for bacteria and *k_sbc_* is the half-saturation constant for the bacterial consumption of *SBC* (in mgC m^−3^).

Bacteria do not assimilate all of the DMSPd they consume, but take only the C and S they need to sustain their growth. It is known that 75 to 90% of DMSPd consumed by bacteria is degraded via demethylation and, although only 5 to 30% of metabolized DMSPd is assimilated into bacterial proteins, and this incorporation could satisfy the total S demands and between 1% and 15% of the C demands of the bacterioplankton [35], [75], [79], [80], [81]. In the model, the bacterial S need (*Sneed*, flux_5_, Fig. 1) is estimated according to their growth, 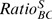, and the bacterial S:C ratio, according to:
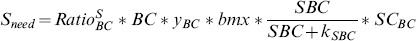
(4)where *y_BC_* is the bacterial growth efficiency and *SC_BC_* is the bacterial S:C ratio (Table 1).

The DMSPd not assimilated is demethylated (1- *lyase_Bact_*, flux_6_, Fig. 1) to produce SO_4_^2−^ or MeSH or cleaved by bacterial DMSP-lyase (*lyase_Bact_*, flux_7_, Fig. 1) as DMS and acrylate and used for the C requirements of the bacteria [35] according to: 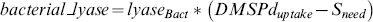
(5)where *lyase_Bact_* is the fraction of DMSPd consumed by bacteria which is cleaved in DMS and fixed to 10% for the reference simulation based on Niki et al. [34]. If DMSPd concentration is not sufficient to support bacterial S needs, DMS can be used as S source (Eq. 7) and bacterial DMSP-lyase activity is null.

Beside bacteria, several studies [82], [83], [84] have shown the capacity of some low DMSP-producer phytoplankton taxa to take up DMSPd. Hence, in parallel to their role of DMSP-producer, phytoplankton could also be a sink for DMS(P) cycle and therefore modify atmospheric DMS emission. However, knowledge on the DMSP-uptake phytoplankton taxa, its ecological role and governing factors and the phytoplankton competitive ability for DMSP regarding bacteria uptake is today insufficient for a proper inclusion in the model.

#### DMS production and fate

DMS is produced from enzymatic cleavage of DMSP by phytoplankton [11] and bacteria [85]. The major loss pathways of DMS are the bacterial consumption via the DMS monooxygenase and methyltransferase and oxidation via the DMS dehydrogenase [7], [68], [86], [87], [88]. DMS is also released to the atmosphere [39], [40] or photooxidized into dimethylsulfoxide (DMSO) [37], [38]. The DMS (in mmolS m^−3^) state equation is:



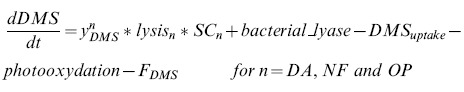
(6)


where 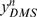 corresponds to the fraction of DMSPp directly cleaved in DMS by phytoplankton DMSP-lyases (flux_1_, Fig. 1), *lysis_n_* is the phytoplankton cellular lysis (in mgC m^−3^ h^−1^), *bacterial_lyase* is the enzymatic cleavage of DMSPd in DMS by bacteria (flux_7_, Fig. 1), *DMS_uptake_* is the bacterial consumption of DMS (flux_8_, Fig. 1), *photooxidation* term is the photochemical oxidation of DMS into DMSO (flux_10_, Fig. 1) and *F_DMS_* is the emission of DMS to the atmosphere through the air-sea water interface (in mmolS m^−3^ h^−1^) (flux_9_, Fig. 1).

Although bacterial degradation of DMS is important [36], [41], [81], [88] less than 10% of S of DMS consumed is incorporated into bacterial biomass [81], [89] and satisfies 1% to 3% of the bacterial S demand. This suggests that DMS is a minor source of S for bacterioplankton, and is probably taken up by bacteria only as a supplementary substrate [81]. Bacteria predominantly metabolized DMS into non-volatile sulfur products, DMSO and SO_4_^3-^ [36], [81], [87], [90].

Based on that, we assume that bacterial uptake of DMS (*DMS_uptake_*) will cover the bacteria S needs if *DMSPd_uptake_* (Eq. 3) is not sufficient. In the model, *DMS_uptake_* is described from the consumption of carbon by bacteria and the DMS content of bacterial C substrates, according to:
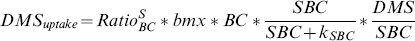
(7)


The photooxidation of DMS into DMSO is described considering a photooxidation constant (*K0*, [91]) modulated by the light extinction coefficient in water, according to:



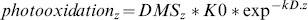
(8)where *z* is the water depth (m), *K0* is the photooxidation rate in the surface (Table 1; [91]) and *kD* is the light extinction coefficient, and (DMS)_Z_ is DMS at depth z. As a first approximation, the ultraviolet A (UVA) penetration in the water column is considered equal to that of photosynthetic active radiation (PAR), as PAR attenuation in the studied coastal area is mainly governed by detrital particulate and colored dissolved organic matter. This assumption corresponds to a maximum water penetration of UVA and tends to overestimate the DMS loss by photooxidation.

The DMS air-sea flux (F_DMS_) is determined based on the surface DMS concentration and the gas transfer velocity (*k*) of DMS at in-situ temperature (*k*_DMS_):
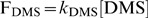
(9)


with



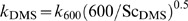
(10)


where k_600_ is *k* normalized to a Schmidt number (Sc) of 600 and Sc_DMS_ is the Sc of DMS computed according to Saltzman et al. [92]:
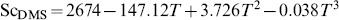
(11)


where *T* is sea surface temperature (°C).

**Figure 2:**
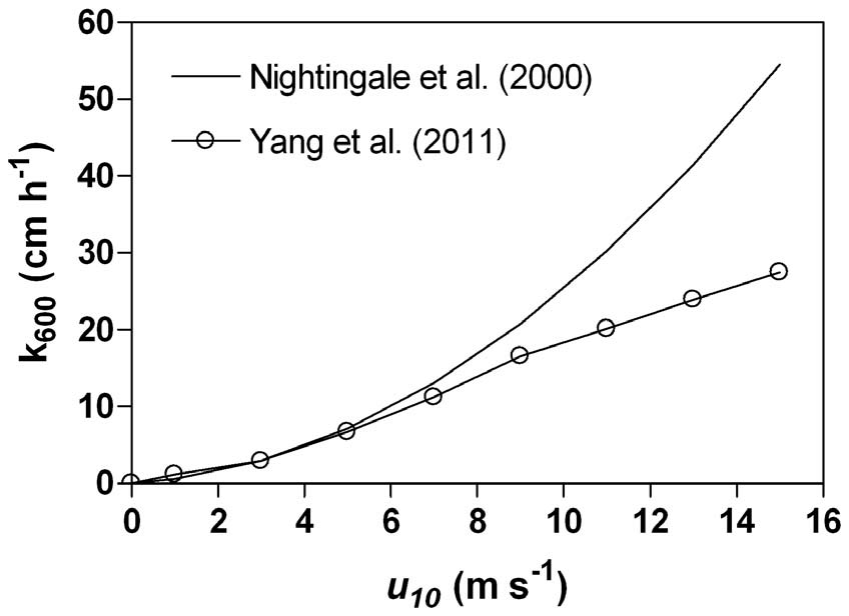
Gas transfer velocity (*k*_600_) as a function of wind speed (u_10_) given by the Nightingale et al. [107] parameterization, and the binned measurements of Yang et al. [93] to which was fitted a polynomial relationship (Eq. 13). The *k* data of Yang et al. [93] were originally reported normalized to a Schmidt number of 660 (*k*_660_) and were converted to *k*_600_.

*k*_600_ (cm h^−1^) was computed from a parameterization (Fig. 2) as a function of wind speed referenced at 10 m height (*u*_10_ in m s^−1^) that we derived from the binned data reported by Yang et al. [93] in their Table 2 (data without bubble normalization):
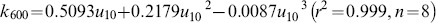
(12)


**Table 2:** F_DMS_ computed for sensitivity tests on DMS(P) model parameters.

	Parameters	Units	Values	Annual mean [DMS]	F_DMS_
				(μmolS m^-3^)	(mmolS m^−2^ y^−1^)
REFERENCE				0.9	0.19
**Sensitivity to phytoplankton parameters**					
Test 1	SC_NF_, SC_OP_	mol S:molC	0.018	1.5	0.32
Test 2	SC_NF_, SC_OP_	mol S:molC	0.004	0.3	0.07
Test 3	SC_DA_	mol S:molC	0.00212	0.9	0.21
Test 4	SC_DA_	mol S:molC	0	0.8	0.18
Test 5	SC_DA_	mol S:molC	0.0034	1.0	0.23
Test 6	y^DA^_DMS_, y^NF^_DMS_, y^OP^_DMS_	-	0	0.5	0.11
Test 7	y^DA^_DMS_, y^NF^_DMS_, y^OP^_DMS_	-	0.25	1.4	0.32
Test 8	y^DA^_DMS_, y^NF^_DMS_, y^OP^_DMS_	-	0.5	2.4	0.53
Test 9	y^NF^_DMS_, y^OP^_DMS_	-	0.5	2.3	0.51
Test 10	y^DA^_DMS_	-	0.5	0.9	0.21
**Sensitivity to bacteria parameters**					
Test 11	SC_BC_	mol S:molC	1:37	0.8	0.17
Test 12	SC_BC_	mol S:molC	1:196	0.9	0.2
Test 13	Ratio_BC_	-	0.75	1.1	0.24
Test 14	Ratio_BC_	-	0.5	1.4	0.32
Test 15	Ratio_BC_ for DMSPd	-	0.5	0.9	0.2
Test 16	Ratio_BC_ for DMS	-	0.5	1.4	0.32
Test 17	khydrolysis	-	0.25	1.6	0.35
**Sensitivity to wind speed and k parameterization**					
Test 18	wind forcing	m s^−1^	3.9	0.9	0.24
Test 19	wind forcing	m s^−1^	−25%	0.9	0.13
Test 20	wind forcing	m s^−1^	+25%	0.9	0.26
Test 21	k parameterization	cm h^−1^	Nightingale	0.9	0.19
			et al., 2002		

*u*_10_ data were extracted from the National Centers for Environmental Prediction (NCEP) Reanalysis Daily Averages Surface Flux (http://www.cdc.noaa.gov/) for one station in the North Sea (3.75°E 52.38°N).

### Model implementation

**Figure 3:**
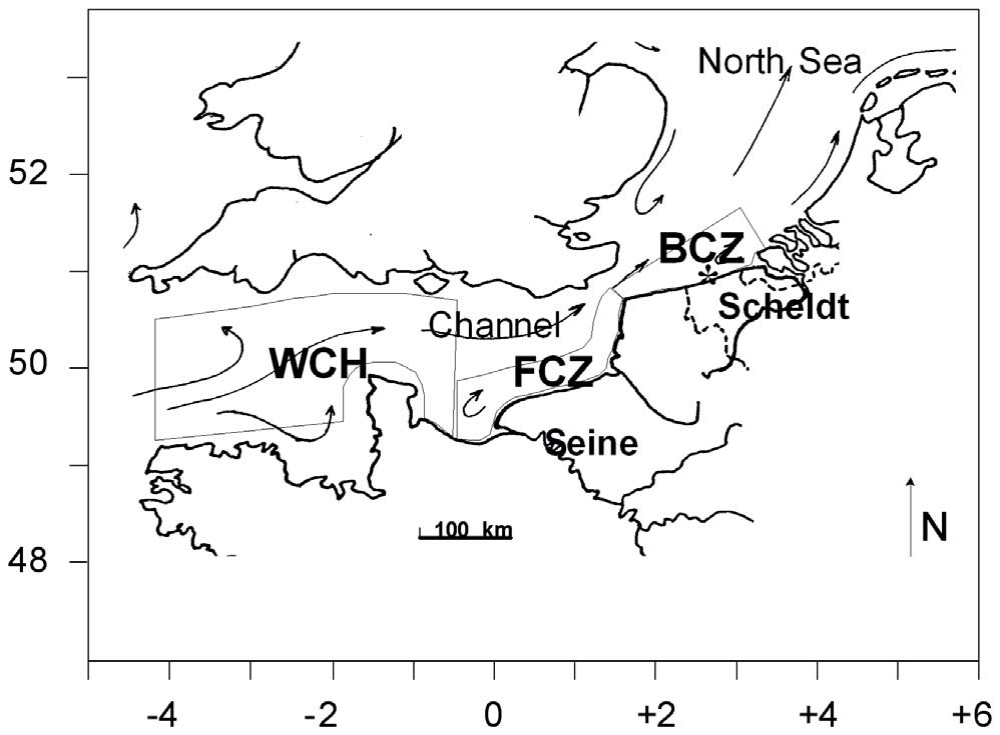
Map of the study area with the MIRO-DMS multi-box frame delimitation with WCH  =  Western Channel; FCZ  =  French Coastal Zone; BCZ  =  Belgian Coastal Zone (adapted from Gypens et al. [94]). Model results analysis will focus on the BCZ where simulated results were daily-averaged for year 1989, when DMS(P) field data are available for comparison.

For this application, the MIRO-DMS model was implemented in the SNS using a multi-box 0D frame delineated on the basis of the hydrological regime and river inputs (Fig. 3) [60]. In order to take account for the cumulated nutrient enrichment of Atlantic waters by the Seine and Scheldt rivers, the model was run successively in the Western Channel (WCH) area considered as a quasi-oceanic closed system, the French coastal zone (FCZ) influenced by the Seine and Atlantic waters from the WCH, and, finally, in the Belgian coastal zone (BCZ) influenced by the direct Scheldt loads and the inflowing FCZ waters. Model simulations were performed using meteorological and river forcing for the year 1989 when DMS(P) data are available for comparison [95]. The seasonal variation of the state variables was calculated by solving the different equations expressing mass conservation according to the Euler procedure. A time step of 15 min was adopted for the computation of the numerical integration. The analysis of daily-averaged model results will be performed in the BCZ where field DMS(P) are available [95]. DMS(P) data for the year 1989 were retrieved from the Global Surface Seawater Dimethylsulfide (DMS) Database (available at http://saga.pmel.noaa.gov/dms/) and correspond to data available in the SNS between 51.0°N–52.5°N and 1.5°E–4.5°E [95].

## Results

### DMS(P) seasonal cycle in the Southern North Sea

Validation of the MIRO ecological model is given by Lancelot et al. [60] and Gypens et al. [69], and is not repeated here. The performance of the MIRO-DMS model is evaluated through its ability to reproduce the seasonal variations of available field data of DMSPp, DMSPd and DMS in the BCZ for the year 1989 [95]. However, due to the limited data set, a statistical validation was not attempted and we only compared qualitatively field data and model output. For this comparison, daily simulated results are compared to data of DMS(P) acquired by Turner et al. [67] during short 2–3 day cruises at monthly intervals. Data for each month ranged between 2 and 15 samples, for the purpose of the validation, they were averaged, and standard deviations are given in plots as error bars.

**Figure 4:**
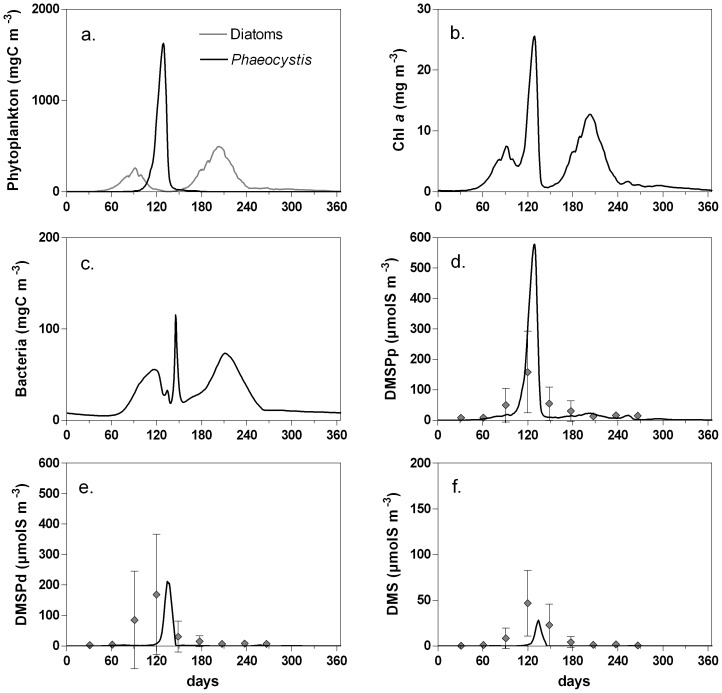
Seasonal evolution of diatoms and *Phaeocystis* colonies biomass (a), total chl a (b), bacteria biomass (c) and DMSPp (d), DMSPd (e) and DMS (f) concentration simulated for year 1989 in the Belgian Coastal Zone by the MIRO-DMS model and compared to monthly DMS(P) averaged data (◊) from Turner et al. [95]. The error bars represent the standard deviation of the mean.

Changes in DMS(P) concentrations are analyzed in parallel to the evolution of the planktonic compartments (phytoplankton and bacteria) (Fig. 4). The phytoplankton evolution simulated in the area is characterized by a succession of spring diatoms, *Phaeocystis* colonies, and summer diatoms (Fig. 4a). Spring diatoms initiate the phytoplankton bloom in early March and are followed by *Phaeocystis* colonies which reach Chl *a* concentration of 25 mgChl*a* m^−3^ (Fig. 4b) in April. Summer diatoms bloom after the *Phaeocystis* decline and remain until fall. On an annual scale, diatom and *Phaeocystis* biomass are similar, the latter being however concentrated during a short period of time, of 1 month (Fig. 4a). In association with the decline of the different phytoplankton blooms, three bacterial maxima are simulated (Fig. 4c).

In agreement with available data, the simulated DMS(P) concentrations show low values except during the spring *Phaeocystis* bloom (Fig. 4d). Simulated DMS(P) values are lower than observed DMS(P) concentrations in early April (the spring diatom bloom). As observed by Turner et al. [67], Kwint and Kramer [68] and van Duyl et al. [96] in North Sea coastal waters, DMSP and DMS concentrations increase in spring and decrease in autumn to low winter values. The maxima in DMS(P) concentrations are limited to a period of about 6 weeks (April, May) and concurred with the *Phaeocystis* bloom as also observed by Stefels et al. [33] in the same area. The model correctly reproduces the observed DMSP seasonal pattern, in particular the timing of the seasonal peak. However, the model fails to reproduce amplitude of the seasonal cycle, with simulated maximal DMSPp concentration (580 µmolS m^−3^; Fig. 4d) three times higher than measured concentration. On the other hand, the modeled DMSPd is much lower than the field observations. This could be due to an experimental bias in older data-sets due to cell breakage leading to an over-estimation of DMSPd and an underestimation of DMSPp [97]. Indeed, the maximum simulated total DMSP (DMSPt  =  DMSPp + DMSPd) of 670 µmolS m^−3^ is close to the maximum observed DMSPt of 730 µmolS m^−3^. This discrepancy could also be due to the low temporal resolution of observations (1 month, [95]), i.e. insufficient to fully capture the dynamics of the system. Indeed, data obtained with a higher sampling frequency (2 samples per week) in the Wadden Sea (Marsdiep) in 1995, show DMSPp concentrations of about 1700 µmolS m^−3^ during a *Phaeocystis* bloom that reached a maximum of 80 10^6^ cell L^−1^ [96]. In agreement with these observations, the simulated maximum of DMSPp (Fig. 4d) coincides with the *Phaeocystis* colonies bloom (Fig. 4a) and reach a value of about 580 µmolS m^−3^ for a *Phaeocystis* biomass of 1600 mgC m^−3^ (Fig. 4a) corresponding to 58 10^6^ cell L^−1^. Hence, the modeled DMSP seasonal peak is bracketed by the lower values of Turner et al. [95] in the more open water of the SNS and the higher values of van Duyl et al. [96] in the near-shore coastal waters of the SNS.

The time lag of about 10 days between the simulated DMSPp and DMSPd (210 µmolS m^−3^; Fig. 4e) peaks is due to the fact that DMSPd results from the phytoplankton lysis and grazing by zooplankton that increase at the end of the bloom. As for DMSPp, simulated DMSPd is also underestimated in comparison with the observed concentration in March during the spring diatom bloom.

The simulated DMS peak reaches a value of 28 µmolS m^−3^ (Fig. 4f) and appears in between DMSPp and DMSPd maxima. The accumulation of DMS simulated during the decay of *Phaeocystis* (Fig. 4a) is consistent with the work of Stefels and van Boekel [61] showing that phytoplankton lyases are active during the stationary phase of the bloom. Simulated and observed DMS show similar seasonal patterns but simulated concentration of DMS is lower than the maxima observed in May (50 µmolS m^−3^, Fig. 4f). However, when spatially averaged over the SNS to take into account for the non-regular distribution of sampling stations, observed DMS concentrations show a maximal value of 25 µmolS m^−3^ (Fig. 5 in Turner et al. [95]).

**Figure 5:**
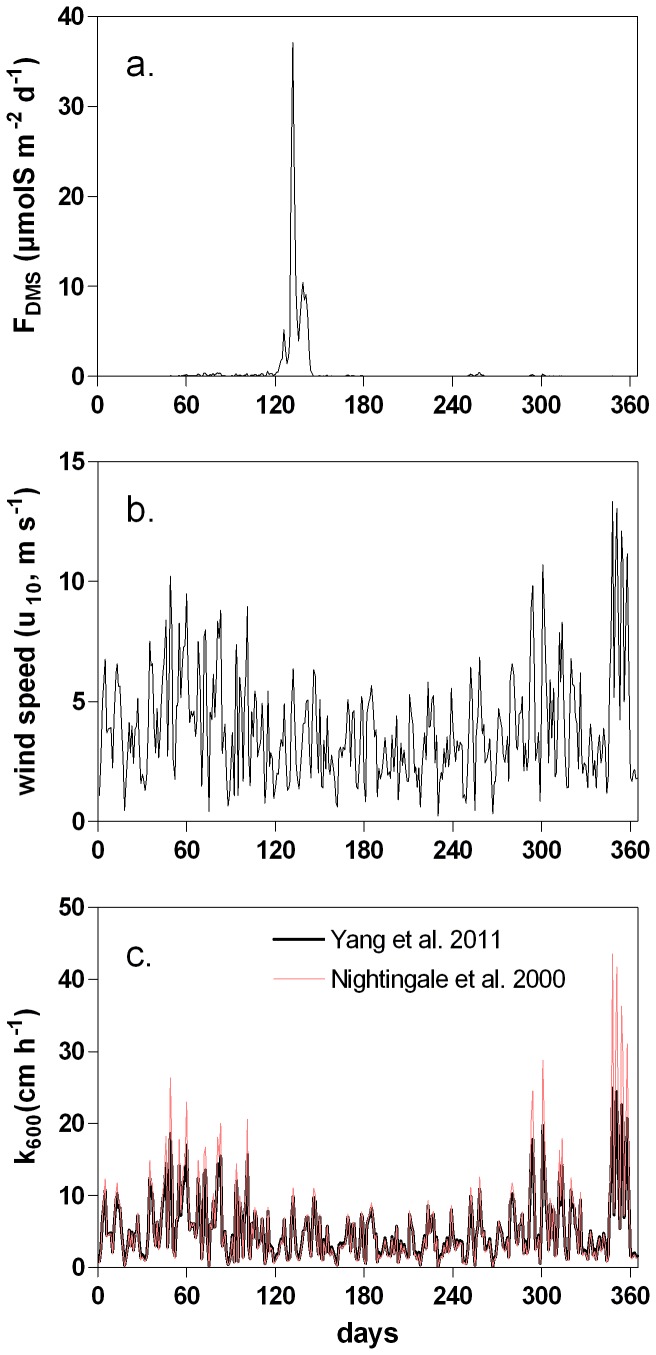
Daily DMS emission (μmolS m^−2^ d^−1^) computed by the MIRO-DMS model in the Belgian Coastal Zone for year 1989 (a), wind speed (u_10_) (b) and the gas transfer velocity (*k*_600_) computed using the Yang et al. [**93**] and the Nightingale et al. [107] relationships (c).

Figure 5a shows the seasonal evolution of atmospheric DMS emissions simulated by the model in the BCZ. As expected the DMS flux to the atmosphere follows closely the temporal pattern of the simulated DMS concentrations (Fig. 4f), ranging from low values in winter to a maximal value of 37 µmolS m^−2^ d^−1^ in spring. The important daily variability simulated during F_DMS_ peak (Fig. 5a) results from wind speed variability (Fig. 5b).

### Annual DMS budget

**Figure 6:**
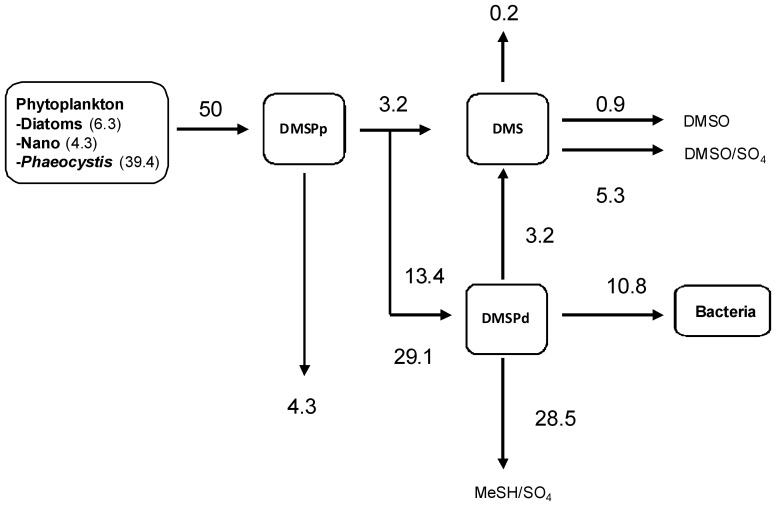
Annual sulfur budget in the Belgian Coastal Zone computed by the MIRO-DMS model for the year 1989 (mmolS m^−2^ y^−1^).

The relative importance of each processes involved in the DMS cycle was estimated based on the annual S budget (Fig. 6) obtained by integrating the daily S rates simulated by the model in the BCZ (Fig. 2) and integrated on the average depth of the study area (17 m).

MIRO-DMS estimates the total annual phytoplankton production of DMSPp at 50 mmolS m^−2^ y^−1^, of which 13% are produced by diatoms, 9% by nanoflagellates and 78% by the *Phaeocystis* colonies. From this, 3.2 mmolS m^−2^ y^−1^ of DMSPp are directly converted in DMS by phytoplankton DMSP-lyase (mainly that of *Phaeocystis*) representing a DMS flux similar to bacterial DMSP-lyase activity. The importance of phytoplankton DMSP-lyase was previously reported in the area by Stefels and Dijkhuizen [25] and Wolfe and Steinke [22]. The production of DMS by phytoplankton DMSP-lyase simulated in the model is three times higher than the DMS loss due to flux to the atmosphere and photochemical oxidation, as observed (between 1.5 to 4.5 times) in the Dutch coast during a *Phaeocystis* bloom [33].

DMSPd results from phytoplankton cell lysis (68%) and zooplankton grazing (32%). The dominant process is the cell lysis of *Phaeocystis*, which in itself releases almost 50% of DMSPp throughout the year. The sedimentation of DMSPp amounts to 4.3 mmolS m^−2^ y^−1^. Bacterial uptake accounts for the majority the removal of both DMSPd and DMS inducing a rapid decrease of their concentrations in the water column. The consumption of DMSPd is sufficient to sustain the total bacteria S need (10.8 mmolS m^−2^ y^−1^), and provides up to 16% of bacteria C requirements. In agreement with previous findings [35], [75], [80], [98], the major fate for simulated DMSPd is the demethylation/demethiolation pathways that consumes 28.5 mmolS m^−2^ y^−1^ and results in S products other than DMS (mainly SO_4_^2−^ and MeSH). Although only 8% (3.2 mmolS m^−2^ y^−1^) of the DMSPd consumed by bacteria is cleaved to DMS, this flux represents 50% of annual DMS input and is similar to phytoplankton DMSP-lyase activity (Fig. 6).

Bacteria also consume directly DMS and about 83% (5.3 mmolS m^−2^ y^−1^) of the DMS pool is consumed by bacteria and transformed in SO_4_^2−^ or DMSO. Kiene and Bates [41] found that microbial DMS consumption was generally 10 times faster than the flux of DMS to the atmosphere. This ratio is about 17 times in our model results with about 14% of the DMS converted into DMSO by photooxidation and finally only 3% of the DMS emitted to the atmosphere. Annual F_DMS_ represents <1% of the DMSPp production in the water column in agreement with Archer et al. [49].

## Discussion

Annual S budget simulated in the BCZ points both phytoplankton and bacteria as key controlling factors of the DMS production. However the relative importance of these processes will results from their description and parameterization in the model. Sensitivity analyses were then carried out to estimate the impact on the atmospheric emission of DMS of the description of several biological processes compared to physical processes (wind speed and *k*_600_ parameterization). In particular, the impact of phytoplankton S:C quota determining the maximal DMSP production of the ecosystem, the importance of phytoplankton DMSP-lyase that represents the direct transformation pathway of DMSP into DMS and the DMS(P) bacterial uptake and lyase activity were tested.

### Sensitivity to biological processes

#### Sensitivity to phytoplankton parameters

**Figure 7:**
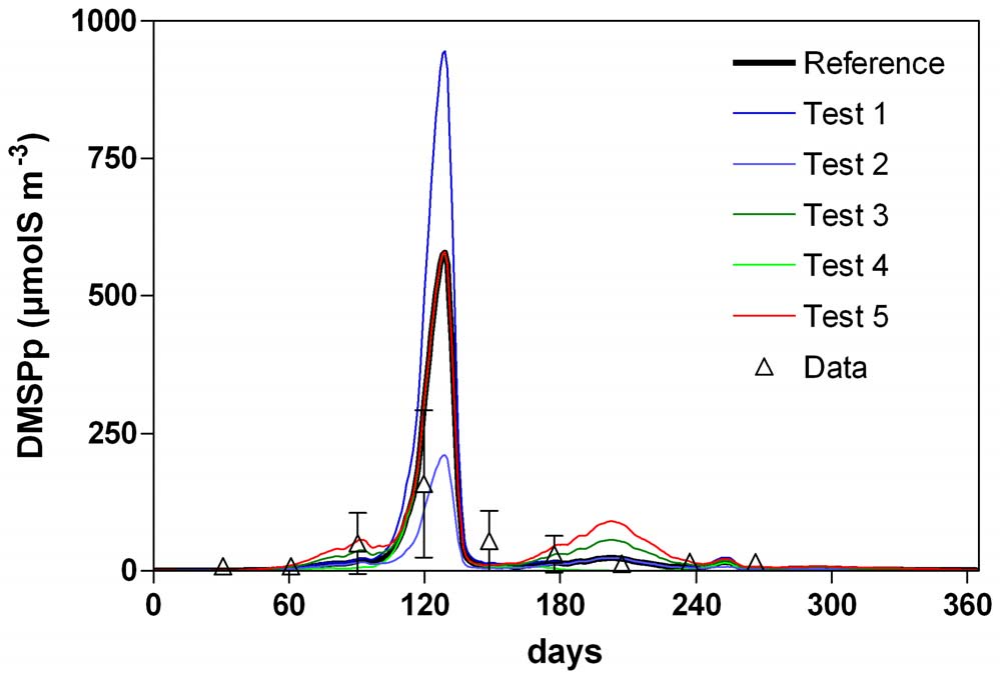
Seasonal evolution of DMSPp concentration simulated by the MIRO-DMS model for year 1989 for different phytoplankton *S:C* ratio (see Table 2 for the description of the sensitivity tests).

**Figure 8:**
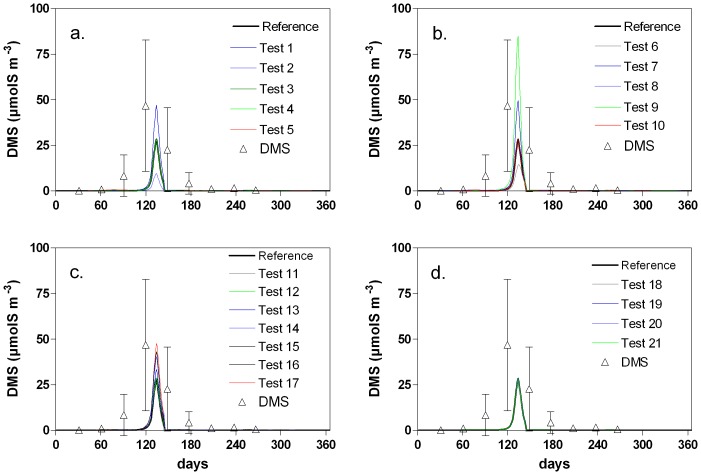
Seasonal evolution of DMS concentration simulated by the MIRO-DMS model for year 1989 by modifying phytoplankton S:C ratio (a), phytoplankton lyase (b), bacteria S:C content and bacterial processes (c) and wind speed (d). See Table 2 for the description of the sensitivity tests.

In our model, phytoplankton S:C quotas were fixed, corresponding to the mean values of measurements for Haptophyceae and diatoms reported by Stefels et al. [7]. Sensitivity tests were performed by varying the S:C quotas within the range of extreme values reported for each phytoplankton type by Stefels et al. [7] (Table 2). Increasing (decreasing) by 70% the *Phaeocystis* S:C value in the model (Test 1 and 2, Table 2) increases (decreases) simulated DMSP and DMS concentrations (Fig. 7; 8a) and annual F_DMS_ by a similar factor (Table 2) without changing the seasonal pattern. Due to the low value of the tested diatom S:C (Tests 3 and 4; Table 2), any modification has little effect on the simulated DMS(P) (Fig. 7; 8a) and F_DMS_ (Table 2). However, some diatom species are characterized by higher S:C quota [8] as *Skeletonema costatum* that is characteristic of the spring diatoms in the SNS [65]. An additional simulation was performed using S:C quota measured for this species (Test 5; Table 2). Increasing the diatom S:C quota increases annual F_DMS_ (Table 2) but also results in an overestimation of simulated DMSPp in summer (Fig. 7). This suggests that in the SNS, dominant diatoms in spring and summer are characterized by different S:C quotas, and that it is essential to take into account for their specific phytoplankton DMSP content to correctly reproduce seasonal evolution of DMS(P) concentration for different FTs (diatoms versus *Phaeocystis*), but also within a FTs (spring versus summer diatoms).

One of the indirect consequences of the choice of the phytoplankton S:C is the possibility for bacteria to fulfil their S need from the consumption of DMS(P). For low phytoplankton S:C ratio (Tests 2 and 4, Table 2) only 60 to 90% of the bacterial S needs in summer and fall can be sustained by DMS(P). As a consequence, the associated bacterial DMSP-lyase activity is decreased.

In the reference simulation, 10% of the DMSP released after phytoplankton lysis is directly cleaved into DMS leading to a DMS flux (3.2 mmolS m^−2^ y^−1^) similar to the DMS flux that comes from bacterial enzymatic cleavage (Fig. 6). However, the relative importance of both processes varies during the seasonal cycle with maximal phytoplankton DMSP-lyase activity simulated at the maximum of the *Phaeocystis* bloom and bacterial DMSP-lyase activity dominating at the decline of the bloom. As deduced by Stefels et al. [7] from the observations of van Duyl et al. [96] in the North Sea, algal DMSP-lyase activity is more important than bacterial enzymatic cleavage at high concentration of DMSPd and explains the occurrence of maximum DMS concentration before the DMSPd peak in our results (Fig. 4e,f). After the decay of the *Phaeocystis* bloom, bacteria and associated DMSP cleavage largely increase.

Most, but not all [34], DMSP-producing species of phytoplankton have DMSP-lyase activity. However, the importance of this activity is not especially correlated with intracellular DMSP concentration [72], [99]. The importance of the direct transformation of DMSP into DMS, on the DMS emission was tested by varying the cleavage yield (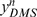, Eq. 3) between 0% and 50% (Table 2). The absence of phytoplankton DMSP-lyase activity (Test 6, Table 2), delays the DMS peak by a few days, and decreases both the simulated DMS (Fig. 8b) and F_DMS_ by about 40% (Table 2). This is higher than the 25% computed by van den Berg et al. [46] based on a modeling study in the SNS. When 25% or 50% of DMSPd released from phytoplankton lysis is converted into DMS (Tests 7 to 9; Table 2), the DMS concentration and F_DMS_ largely increase compared to the reference simulation (from 1.5 to 2.5 times, Table 2). Although simulated DMSPd decreases, this effect is limited as the DMSPd pool is also provided by zooplankton grazing, and its fate controlled by bacterial activity. Increasing only diatom DMSP-lyase yield has little effect on F_DMS_ (Test 10; Table 2), indicating the dominance of *Phaeocystis* in phytoplankton DMSP-lyase activity.

Altogether these sensitivity tests show that phytoplankton DMSP-lyase is a key process controlling both DMS concentration and F_DMS_ and even more important when associated to a high DMSP-producer such as *Phaeocystis*. It is therefore important to determine this enzymatic activity in high DMSP-producing species or among species that co-occur with high DMSP-producing species. An explicit description of DMSP-lyase activity in models could also be important if this activity varies as a function of environmental conditions.

#### Sensitivity to bacteria parameters

As observed by several authors (e.g. [75]), bacterial uptake is the major fate of DMSPd in the model, but only 8% of this DMSPd is cleaved into DMS by bacteria. This agrees with recent observations concluding that bacteria are not key players in DMSPd cleavage into DMS [100], [101] but play a major role in regulating the flux of DMS indirectly by the consumption and demethylation of DMSPd with production of S product other than DMS.

However, the proportion of DMSPd consumed by bacteria and transformed into DMS is function of the DMSPd concentration [96] and the bacterial S demand [35]. Indeed, previous studies suggested that the fraction of DMSPd converted into DMS increases with DMSPd concentration [75]. Lower DMSPd concentrations are completely assimilated, whereas higher concentrations result in increasing amounts of DMS produced [102]. Moreover, a strong demand for S decreases bacterial cleavage of DMSPd [35]. The sensitivity of model results to DMSPd concentration and/or bacterial S needs was estimated either by modifying the release of DMSPd by phytoplankton, the bacterial S:C quota or the proportion of the bacterial community that use DMSP as S source.

In the model, DMSPd is released in the water column by phytoplankton lysis and grazing processes (Eq. 2). The modification of the phytoplankton DMSP-lyase activity affects the F_DMS_ but also the relative contribution of phytoplankton and bacterial processes to DMS production. Increasing the direct transformation of DMSPp in DMS by phytoplankton DMSP-lyase will decrease the DMSPd bacterial uptake and the bacterial production of DMS. Increasing the cleavage yield (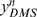, Eq. 3) up to 50% (Test 8, Table 2) will decrease bacterial DMS production by 40% but increases both DMS production by phytoplankton and F_DMS_. However, when compared to the data available in the literature [7], [34], these results overestimate the contribution of phytoplankton compared to bacteria to the DMS production (with phytoplankton contribution up to 90% of the DMS production). One possible source of overestimation of DMSPd concentrations in the model can however result from the assumption that all the DMSPp ingested by micro- and meso-zooplankton is transformed into DMSPd (Eq. 1, 2). Indeed, Wolfe and Steinke [22] also suggested that part of the DMSPp can be directly converted to DMS since digestion promotes the activity of DMSP-lyase present in the membrane of the prey. To test the impact of the direct conversion of DMS by zooplankton, 30% of DMSPp (based on Archer et al. [74]) ingested by grazing was directly transformed in DMS and added in Eq. 6. This results in an increase of DMS concentration in the water column and of F_DMS_ (0.29 mmolS m^−2^y^−1^) with little impact on DMSPd concentration.

Sensitivity tests were then conducted by varying the bacterial S:C quota between extreme values reported in the literature i.e. 1∶37 and 1∶196 molS:molC ([103]; Tests 11 and 12; Table 2). In our model, decreasing the bacterial S:C ratio will decrease the proportion of consumed DMSPd that will be assimilated by bacteria and increase the cleavage of DMSPd into DMS. This can enhance the F_DMS_. However, as shown in Table 2, this parameter is not very sensitive in our application as the DMSPd produced is largely enough to fulfil the S needs of the whole bacterial community.

In a third series of tests, we modified the percentage of bacteria able to use DMSPd and/or DMS as S source. The hypothesis of 100% used in the reference simulation was based on the observation that most marine bacteria have the genetic capability to demethylate DMSP [36], [104], [105] and that DMSPd/DMS concentrations can support almost all bacteria S needs [79], [80], [87]. However, all bacteria are not able to metabolize DMSP and/or DMS. We therefore explore the sensitivity of the model to the bacteria diversity by decreasing this proportion to 75% or 50% (Tests 13 and 14; Table 2). As expected, the turnover rate of DMSPd and DMS decreases and the F_DMS_ increases (Table 2). The maximum concentrations of DMSPd and DMS (Fig. 7c) simulated are 270 µmolS m^−3^ and 33 µmolS m^−3^ when considering that 75% of the bacterial community is able to degrade DMS(P) and 375 µmolS m^−3^ and 40 µmolS m^−3^ for a fraction of 50%. The simulated DMS emissions to the atmosphere also increase with an annual F_DMS_ of about 0.24 and 0.32 mmolS m^−2^ y^−1^, respectively, compared to 0.19 mmolS m^−2^ y^−1^ in the reference simulation (Table 2). This increase of DMS emission results from the combination of bacterial DMSP cleavage and the decrease of bacterial DMS uptake. In these simulations, bacterial DMSP-lyase activity shows a small increase (up to 3.4 and 3.6 mmolS m^−2^ y^−1^ compared to 3.2 mmolS m^−2^ y^−1^ in the reference simulation), and the increase of F_DMS_ mainly results from the decrease of bacterial DMS uptake and the accumulation of DMS in the water column. This is confirmed by results obtained by modifying only DMSPd (Test 15) or DMS bacterial uptake (Test 16). These results are consistent with the observations that suggest that bacterial DMS uptake may be a quantitatively important sink for DMS from the surface ocean [36], [81], [87], [90].

In the model, bacterial cleavage of DMSP in DMS represents 10% of the uptake of DMSPd not assimilated by bacteria. To test the importance of bacterial DMSP-lyase activity, this fraction was set to 25% inducing an increase of almost two fold of both the concentration of DMS and F_DMS_.

Due to their importance on both DMSPd and DMS transformation, bacterial processes need to be accurately described and/or parameterized in ecosystem models. Note that in the present version of the model we only considered one bacterial community, and we did not individually represent the DMS- or DMSP-consumers although this simplification also induces possible uncertainties and underestimation of F_DMS_ resulting from the maximal hypothesis of bacterial uptake (Ratio^BC^_S_ = 1). This is particularly important for the direct bacterial uptake of DMS. Similarly, the bacteria state variable lumps both bacteria and Achaea that might also be important for the demethylation/demethiolation processes [106].

### Sensitivity to physical processes: Wind speed and *k*_600_ parameterization

Besides biological processes, F_DMS_ is also function of the *k*_600_ that depends on the intensity of wind speed and how it is translated into turbulence (depending on the parameterization). Additional tests were performed to estimate the sensitivity of the simulated atmospheric emission of DMS to wind speed and *k*_600_ parameterization (Table 2). Changing wind speed will mainly affect the F_DMS_ that change up to 37% (Test 20, Table 2) with little change for DMS concentrations (Fig. 8d). Due to very low values of wind speed (Fig. 5b) during the *Phaeocystis* bloom and the peak production of DMS (Fig. 4a,d), the use of a constant annual mean wind speed will increase annual F_DMS_ (Test 18; Table 2). Indeed, to accurately compute F_DMS_ it is required to use high temporal resolution *u*_10_ data [49]. However, considering the low effect of F_DMS_ compared to bacterial DMS consumption, this has little impact on the dissolved DMS concentration (Fig. 8d).

In the reference simulation we used a parameterization of *k*_600_ based on the data reported by Yang et al. [93]. Several other parameterizations of *k*_600_ exist and for the purpose of a sensitivity analysis, we chose the one of Nightingale et al. [107] that has been used in the recent F_DMS_ climatology of Lana et al. [40]. Nightingale et al. [107] parameterize *k*_600_ as a function of *u*_10_, according to:
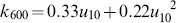
(13)


The *k* values used in the Nightingale et al. [107] parameterization were determined from two dual tracer (^3^He and SF_6_) release experiments in the SNS, and this parameterization has been shown to be also applicable in open ocean conditions [108]. The *k* values of Yang et al. [93] were obtained from measurements of [DMS] and direct measurements of F_DMS_ by eddy-covariance during 2 experiments in the Pacific Ocean and 3 experiments in the Atlantic Ocean. The *k*_600_ values of Nightingale et al. [107] and Yang et al. [93] strongly diverge at u_10_>8 m s^−1^ (Fig. 2). This has been attributed to reduced bubble-mediated transfer at high wind speeds of highly soluble DMS compared to enhanced bubble-mediated transfer of sparingly soluble gases such as ^3^He and SF_6_.

The net annual F_DMS_ computed with the Yang et al. [93] derived parameterization (Eq. 12) and the Nightingale et al. [107] parameterization (Eq. 13) are not different in the area during the simulation period. This is due to the fact that during the period of high DMS concentrations (during the *Phaeocystis* bloom) wind speed is low (average 3.3±1.7 m s^−1^, Fig. 5b), and the *k*_600_ values computed from the two relationships are very close (Fig. 5c). The two *k*_600_ relationships only significantly diverge for *u*_10_>8 m s^−1^ (Fig. 2), and such *u*_10_ values only occur during winter and fall in the SNS (Fig. 5b) when [DMS] is very low or zero (Fig. 4f). Since wind speeds>8 m s^−1^ are rare events in the area (<6% of observations), the annual average of *k*_600_ computed from the Yang et al. [93] relationship (5.20 cm h^−1^) is only ∼9% lower than the one computed using the Nightingale et al. [107] relationship (5.67 cm h^−1^).

### Comparison of DMS and F_DMS_ modelled by the mechanistic MIRO-DMS model and derived from empirical relationships (statistical models)

**Figure 9:**
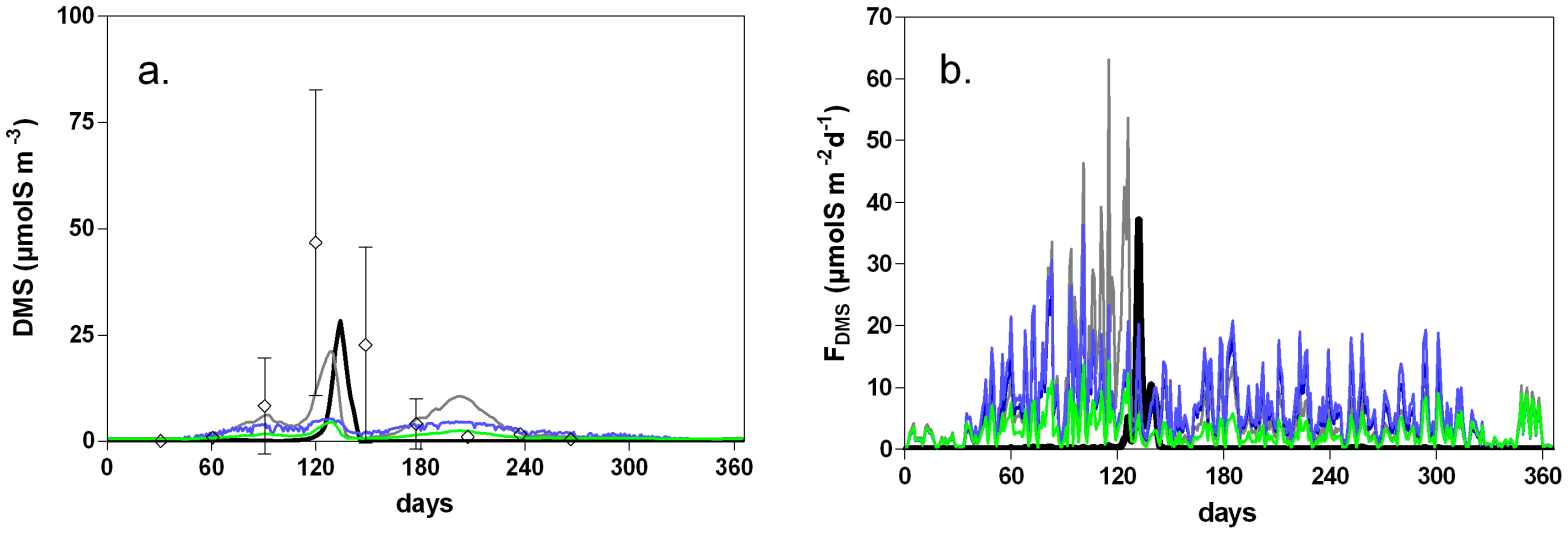
Seasonal evolution of DMS concentration (a) and flux (b) computed in the BCZ for year 1989 using the MIRO-DMS model (black) and the empiric relationship of Simó and Dachs [111] (grey), Anderson et al. [109] with a kNO3 of 0.8 and 2 mmolN m^−3^ (blue) and Lana et al. [114] (green) and compared to available data (◊) from Turner et al. [95]. The error bars represent the standard deviation on the mean.

In order to achieve global [109], [110], [111], [112], [113], [114], [115] or regional [116] estimates of F_DMS_, several empirical relationships have been derived from DMS field data and variables such Chl *a*, NO_3_^−^, T, primary production, solar radiation, or mixed layer depth that can be derived at higher spatial and temporal resolution from climatologies, remote sensing or models. We tested if some of these empirical relationships that are assumed universal and generic were applicable to the SNS that is representative of a temperate eutrophied coastal system. Several empirical parameterizations that allow to compute DMS concentration in marine waters (Table 3) were applied in the area using MIRO-DMS outputs (Chl *a*, NO_3_^−^) and compared to DMS concentration obtained with the MIRO-DMS, and with the available DMS observations in area (Fig. 9a).

**Table 3:** Empirical relationships tested in the MIRO-DMS, and the corresponding annual mean of [DMS] and F_DMS_. *Fp* is the community structure index computed as the ratio between the diatoms and non-diatoms (nanoflagellates and *Phaeocystis* colonies) Chl *a* simulated by the MIRO-DMS and *z* in the depth of the mixed layer (m) that is constant in the MIRO-DMS application (17m).

Equations	Reference	[DMS]	F_DMS_
		(μmolS m^−3^)	(mmol S m^−2^ y^−1^)
[DMS] = 2.29 for log10(CJQ)<1.72	Anderson et al. [109]	2.2	2.23 for k_NO3_ = 0.8
[DMS] = 8.24 [log10(CJQ)−1.72]+2.29 for log10(CJQ)>1.72		2.5	2.63 for k_NO3_ = 2
where C = Chl a (mgm^−3^), J = mean daily irradiance (Wm^−2^)			
and Q = NO_3_/(NO_3_+k_NO3_) (mmolm^−3^)			
[DMS] = −ln (z)+5.7 for Chl a/z<0.02	Simó and Dachs [111]	3.1	3.03
[DMS] = 55.8 (Chl a/z)+0. for Chl a/z>0.02			
[DMS] = 2.356+0.614 * Chla	Lana et al. [114]	1.1	1.21
DMSPp = (20*Chla*Fp)+21 for Chla'<0.3 mg m^−3^	Belviso et al. [112]	-	-
DMSPp = (20*Chla*Fp)+(356.4 * Chla −85.5)			
for Chla'>0.3 mg m^−3^			
DMS:DMSP = 0.231−3.038Fp−16 Fp^2^			
−38.05Fp^3^+41.12Fp^4^−16.32Fp^5^			
DMSPp = (20*Chla*Fp)+	Aumont et al. [110]	-	-
(13.64+0.10769* (1+24.97*(1-Fp)*Chla)^2.5^)			
DMS:DMSP = 0.015316+0.005294/(0.0205+Fp) for Fp<0.6			
DMS:DMSP = 0.674*Fp−0.371 for Fp>0.6			

DMS concentrations simulated with the algorithm of Simó and Dachs [111] show maximal DMS concentrations similar to those simulated by the model during the *Phaeocystis* bloom (Fig. 9a). However, they overestimate F_DMS_ along the seasonal cycle (Fig. 9b), in particular due to an overestimation of the DMS concentrations related to spring and summer diatom blooms (Fig. 9a). Neither Anderson et al. [109] nor Lana et al. [114] relationships can reproduce the amplitude of DMS seasonal cycle and DMS peak associated to *Phaeocystis* bloom (Fig. 9a). As for the Simó and Dachs [111] relationship, the Anderson et al. [109] and Lana et al. [114] relationships over-estimate the DMS concentration associated with the diatom spring and summer blooms. In the area, the mixed layer depth is constant ( = total depth, since it is a permanently well-mixed shallow system) and the seasonal evolution of DMS concentrations (Fig. 9a) simulated by all these relationships is controlled by the evolution of Chl *a* (Fig. 9c), without any distinction in DMSP cellular content among phytoplankton groups. To take into account of this variability we also tested two additional relationships respectively developed by Aumont et al. [110] and revised by Belviso et al. [112] based on a similar data-set. The Fp ratio representing the community structure index (and corresponding to the ratio of the diatoms and dinoflagellates to the total Chl *a*) used in these relationship was computed based diatoms and non-diatoms (nanoflagellates and *Phaeocystis* colonies) Chl *a* simulated by the MIRO-DMS model. Results obtained with both relationships largely overestimated DMS concentrations in the area during *Phaeocystis* bloom (with DMS values up to 400 nM with the Belviso et al. [112] equation and unrealistic values up to 5000 nM with the Aumont et al. [110] equation). Both relationships were established from data-sets with total Chl *a* values <4 µg L^−1^ (and non-diatom Chl *a* values lower than 1 µg L^−1^), well below the maximum values in the SNS, up to 25 µg L^−1^ (Fig. 4b). Based on these results, we conclude that these relationships are not adapted to ecosystems dominated by high biomass of non-siliceous species, typically in eutrophied coastal environments.

The F_DMS_ computed from DMS derived the various empirical parameterizations are higher than F_DMS_ computed with MIRO-DMS, about 6 times higher for Lana et al. [114] and about 10 to 15 times higher for Anderson et al. [109] and Simó and Dachs [111] relationships. These F_DMS_ values are also largely higher than the maximal F_DMS_ previously estimated in the area [33], [46], [67], [117]. Despite the fact that these relationships give lower seasonal maxima DMS concentrations (with the exception of the Simo and Dachs [111] relationship), they compute DMS concentrations through the year during both diatom and *Phaeocystis* blooms. MIRO-DMS only simulates DMS during the *Phaeocystis* bloom, when wind speed and *k*_600_ are low (Fig. 5b,c), while DMS is very low during the rest of the year.

## Conclusions

The application in the BCZ of the newly developed biogeochemical model MIRO-DMS shows that modelled F_DMS_ is more sensitive to the description and parameterization of biological than abiotic processes. The results confirm the importance of accounting for specific phytoplankton cellular DMSP between different FTs (*Phaeocystis versus* diatoms) but also within a FT (spring *versus* summer diatoms) to describe DMSP and DMS concentrations in marine ecosystems. Due to their elevated S:C quota and their major contribution (50%) to the annual primary production, *Phaeocystis* colonies are responsible of 78% of the annual production of DMSP in the BCZ. This work is an additional modelling effort to explicitly include bacterial processes in transforming DMS(P), and shows their contribution in processing DMSP and as a sink of DMS that is much higher than DMS removal by photooxidation and F_DMS_.

Current empirical relationships to predict DMS from Chl *a* [109], [110], [111], [112], [114] were unable to satisfactorily reproduce the seasonal cycle of DMS in timing and amplitude in the SNS in comparison with field data and MIRO-DMS simulations. In the data-sets from which these empirical relationships were established, the high Chl *a* values were related to diatoms unlike eutrophied coastal environments such as the SNS where high biomass is not associated to diatoms. Therefore, future projections of F_DMS_ and the investigation of the potential feedback on climate require to use modeling tools that accurately represent DMS(P) dynamics in coastal environments that are hotspots of DMS emissions, in particular, in eutrophied coastal environments dominated by high biomass non-diatom blooms. Further, bacterial processing of DMS(P) needs to be correctly represented in models. The potential feedbacks of DMS emissions on climate will depend on the impact of climate change on the phytoplankton composition and biomass, as postulated by the CLAW hypothesis [1], but also of the response of the bacterial communities to global changes, and how they will modulate the sinks of DMS in seawater (emission to the atmosphere *versus* bacterial consumption/transformation).
