# Correction: The Dimethylsulfide Cycle in the Eutrophied Southern North Sea: A Model Study Integrating Phytoplankton and Bacterial Processes

**DOI:** 10.1371/journal.pone.0105178

**Published:** 2014-08-22

**Authors:** The PLOS ONE Staff

There is an error in Table 3. The authors have provided a corrected version below.

**Table 3:** Empirical relationships tested in the MIRO-DMS, and the corresponding annual mean of [DMS] and F_DMS_. *Fp* is the community structure index computed as the ratio between the diatoms and non-diatoms (nanoflagellates and *Phaeocystis* colonies) Chl *a* simulated by the MIRO-DMS and *z* in the depth of the mixed layer (m) that is constant in the MIRO-DMS application (17m).

Equations	Reference	[DMS]	F_DMS_
		(µmolS m^-3^)	(mmol S m^-2^ y^-1^)
[DMS] = 2.29 for log10(CJQ) < 1.72	Anderson et al. [109]	2.2	2.23 for k_NO3_ = 0.8
[DMS] = 8.24 [log10(CJQ)−1.72]+2.29 for log10(CJQ) > 1.72		2.5	2.63 for k_NO3_ = 2
where C = Chl *a* (mg m^-3^), J = mean daily irradiance (W m^-2^) and Q = NO_3_/(NO_3_+k_NO3_) (mmol m^-3^)			
[DMS] = -ln (z) +5.7 for Chl *a*/z < 0.02	Simó and Dachs [111]	3.1	3.03
[DMS] = 55.8 (Chl *a*/z) + 0.6 for Chl *a*/z > 0.02			
[DMS] = 2.356 + 0.614 * Chl *a*	Lana et al. [114]	1.1	1.21
DMSPp = (20*Chl a*Fp) + 21 for Chl *a*' < 0.3 mg m^-3^	Belviso et al. [112]	-	-
DMSPp = (20*Chl a*Fp) + (356.4 * Chl *a*' -85.5) for Chl *a*' > 0.3 mg m^-3^			
where Chl *a*' = nanophytoplankton Chl *a*			
DMS:DMSP = 0.231 - 3.038Fp +16 Fp^2^ −38.05Fp^3^ + 41.12Fp^4^ −16.32Fp^5^			
DMSPp = (20*Chl a*Fp)+	Aumont et al. [110]	-	-
(13.64 + 0.10769* (1+24.97*(1-Fp)*Chl *a*)^2.5^)			
DMS:DMSP = 0.015316 + 0.005294/(0.0205+Fp) for Fp<0.6			
DMS:DMSP = 0.674 *Fp-0.371 for Fp>0.6			
